# Supplementary material for: Detection of COVID-19 features in lung ultrasound images using deep neural networks
Source: Commun Med (Lond). 2024 Mar 11;4:41. doi: 10.1038/s43856-024-00463-5 (PMC10928066; doi:10.1038/s43856-024-00463-5)
Supplement: Supplementary file 2 — Description of Additional Supplementary Files [file 43856_2024_463_MOESM2_ESM.pdf]

Description of Additional Supplementary Files

File Name: Supplementary Data 1
Description: Source data for figures
