# Supplementary material for: Detection of COVID-19 features in lung ultrasound images using deep neural networks
Source: Commun Med (Lond). 2024 Mar 11;4:41. doi: 10.1038/s43856-024-00463-5 (PMC10928066; doi:10.1038/s43856-024-00463-5)
Supplement: Supplementary file 4 — Reporting Summary [file 43856_2024_463_MOESM4_ESM.pdf]

nature portfolio

Corresponding author(s): Muyinatu A. Lediju Bell

Last updated by author(s): Dec 10, 2023

Reporting Summary

Nature Portfolio wishes to improve the reproducibility of the work that we publish. This form provides structure for consistency and transparency in reporting. For further information on Nature Portfolio policies, see our Editorial Policies and the Editorial Policy Checklist.

Statistics

For all statistical analyses, confirm that the following items are present in the figure legend, table legend, main text, or Methods section.

n/a	Confirmed	
☐	☒	The exact sample size (*n*) for each experimental group/condition, given as a discrete number and unit of measurement
☐	☒	A statement on whether measurements were taken from distinct samples or whether the same sample was measured repeatedly
☐	☒	The statistical test(s) used AND whether they are one- or two-sided<br>*Only common tests should be described solely by name; describe more complex techniques in the Methods section.*
☒	☐	A description of all covariates tested
☐	☒	A description of any assumptions or corrections, such as tests of normality and adjustment for multiple comparisons
☐	☒	A full description of the statistical parameters including central tendency (e.g. means) or other basic estimates (e.g. regression coefficient) AND variation (e.g. standard deviation) or associated estimates of uncertainty (e.g. confidence intervals)
☐	☒	For null hypothesis testing, the test statistic (e.g. *F*, *t*, *r*) with confidence intervals, effect sizes, degrees of freedom and *P* value noted<br>*Give P values as exact values whenever suitable.*
☒	☐	For Bayesian analysis, information on the choice of priors and Markov chain Monte Carlo settings
☒	☐	For hierarchical and complex designs, identification of the appropriate level for tests and full reporting of outcomes
☒	☐	Estimates of effect sizes (e.g. Cohen's *d*, Pearson's *r*), indicating how they were calculated

Our web collection on statistics for biologists contains articles on many of the points above.

Software and code

Policy information about availability of computer code

Data collection	We used Clarius Ultrasound App provided by Clarius Mobile Health Corp. to collect clinical ultrasound image data. The version used was 8.0.1.
Data analysis	Our custom code for training and testing deep learning models were written with Python 3.8. We also used MATLAB to write custom code for analyzing testing results from deep learning models. The MATLAB version we used was MATLAB R2021a.

For manuscripts utilizing custom algorithms or software that are central to the research but not yet described in published literature, software must be made available to editors and reviewers. We strongly encourage code deposition in a community repository (e.g. GitHub). See the Nature Portfolio guidelines for submitting code & software for further information.

Data

Policy information about availability of data

All manuscripts must include a data availability statement. This statement should provide the following information, where applicable:

- Accession codes, unique identifiers, or web links for publicly available datasets
- A description of any restrictions on data availability
- For clinical datasets or third party data, please ensure that the statement adheres to our policy

The web link for our publicly available datasets is: https://gitlab.com/pulselab/covid19.
The restriction on data availability: The dataset acquired in the Emergency Department of Johns Hopkins Hospital was under the Institutional Review Board Protocol that does not allow public data sharing.

Human research participants

Policy information about studies involving human research participants and Sex and Gender in Research.

Reporting on sex and gender	Sex and gender were not considered in study design. Sex- and gender-based analysis was not performed because our research purpose is to investigate the detection of in vivo COVID-19 features in ultrasound B-mode images with deep neural networks trained on customized datasets. There is no reason to believe that the detection ability of these deep neural networks is related to sex or gender. Therefore, we consider sex and gender to be irrelevant to our study.
Population characteristics	There is no covariate-relevant population characteristics of the human research participants in our study because our research purpose is to investigate the detection of in vivo COVID-19 features in ultrasound B-mode images with deep neural networks trained on customized datasets. The detection ability of these deep neural networks is irrelevant to population characteristics.
Recruitment	No patients were recruited because our study is a retrospective study which made secondary use of lung POCUS data collected as part of the standard clinical care of patients with suspected or confirmed COVID-19 infection.
Ethics oversight	Johns Hopkins Medicine Institutional Review Boards

Note that full information on the approval of the study protocol must also be provided in the manuscript.

Field-specific reporting

Please select the one below that is the best fit for your research. If you are not sure, read the appropriate sections before making your selection.

☒ Life sciences ☐ Behavioural & social sciences ☐ Ecological, evolutionary & environmental sciences

For a reference copy of the document with all sections, see nature.com/documents/nr-reporting-summary-flat.pdf

Life sciences study design

All studies must disclose on these points even when the disclosure is negative.

Sample size	Sample size was determined based on the data availability and sample size normally used in the studies of deep learning applications on medical images.
Data exclusions	There were no data exclusions.
Replication	We ran experimental codes several times using the same random seed to verify the reproducibility of the experimental findings. All attempts at replication were successful.
Randomization	Samples were allocated into experimental groups (training dataset and testing dataset in our case) randomly.
Blinding	The investigators were blinded to group allocation during data analysis.

Reporting for specific materials, systems and methods

We require information from authors about some types of materials, experimental systems and methods used in many studies. Here, indicate whether each material, system or method listed is relevant to your study. If you are not sure if a list item applies to your research, read the appropriate section before selecting a response.

Materials & experimental systems

n/a	Involved in the study	
☒	☐	Antibodies
☒	☐	Eukaryotic cell lines
☒	☐	Palaeontology and archaeology
☒	☐	Animals and other organisms
☐	☒	Clinical data
☒	☐	Dual use research of concern

Methods

n/a	Involved in the study	
☒	☐	ChIP-seq
☒	☐	Flow cytometry
☒	☐	MRI-based neuroimaging

Clinical data

Policy information about clinical studies

All manuscripts should comply with the ICMJE guidelines for publication of clinical research and a completed CONSORT checklist must be included with all submissions.

Clinical trial registration	Our study only involves a retrospective data analysis and is not a clinical trial.
Study protocol	The full trial protocol is not available because our study is not a clinical trial.
Data collection	The clinical data was collected in the Emergency Department of Johns Hopkins Hospital. There is no recruitment because this is a retrospective study.
Outcomes	This is not available because our study is not a clinical trial.
